# The Molecular Chaperone HSP90 Promotes Notch Signaling in the Germline of *Caenorhabditis elegans*

**DOI:** 10.1534/g3.118.300551

**Published:** 2018-03-05

**Authors:** James L. Lissemore, Elyse Connors, Ying Liu, Li Qiao, Bing Yang, Mark L. Edgley, Stephane Flibotte, Jon Taylor, Vinci Au, Donald G. Moerman, Eleanor M. Maine

**Affiliations:** * Biology Department, John Carroll University, University Heights, OH 44118; † Department of Biology, Syracuse University, NY 13244; ‡ Department of Zoology, University of British Columbia, Vancouver, BC, Canada V6T 1Z3

**Keywords:** *C. elegans*, HSP90, germline stem cells, Notch, GLP-1

## Abstract

In a genetic screen to identify genes that promote GLP-1/Notch signaling in *Caenorhabditis elegans* germline stem cells, we found a single mutation, *om40*, defining a gene called *ego-3. ego-3(om40)* causes several defects in the soma and the germline, including paralysis during larval development, sterility, delayed proliferation of germline stem cells, and ectopic germline stem cell proliferation. Whole genome sequencing identified *om40* as an allele of *hsp-90*, previously known as *daf-21*, which encodes the *C. elegans* ortholog of the cytosolic form of HSP90. This protein is a molecular chaperone with a central position in the protein homeostasis network, which is responsible for proper folding, structural maintenance, and degradation of proteins. In addition to its essential role in cellular function, HSP90 plays an important role in stem cell maintenance and renewal. Complementation analysis using a deletion allele of *hsp-90* confirmed that *ego-3* is the same gene. *hsp-90(om40)* is an I→N conservative missense mutation of a highly conserved residue in the middle domain of HSP-90. RNA interference-mediated knockdown of *hsp-90* expression partially phenocopied *hsp-90(om40)*, confirming the loss-of-function nature of *hsp-90(om40)*. Furthermore, reduced HSP-90 activity enhanced the effect of reduced function of both the GLP-1 receptor and the downstream LAG-1 transcription factor. Taken together, our results provide the first experimental evidence of an essential role for HSP90 in Notch signaling in development.

The HSP90 molecular chaperone plays critical roles in protein homeostasis (proteostasis), participating in the folding, maturation, and degradation of hundreds of substrate proteins, known as clients (Eckl and Richter 2013; Li *et al.* 2013; Karagöz and Rüdiger 2015; Haase and Fitze 2016; Schopf *et al.* 2017). Metazoans encode several highly conserved HSP90 proteins with specific isoforms localized to mitochondria, chloroplasts, endoplasmic reticulum, and the cytosol (Eckl and Richter 2013; Röhl *et al.* 2013; Haase and Fitze 2016; Schopf *et al.* 2017). Operating in conjunction with more than 20 co-chaperones, the HSP90 homodimer is an ATP-dependent molecular machine that binds to partially folded proteins to assist in their maturation through a yet-to-be-elucidated mechanism (Eckl and Richter 2013; Li *et al.* 2013; Röhl *et al.* 2013; Mayer and Le Breton 2015; Haase and Fitze 2016; Pearl 2016; Bar-Lavan *et al.* 2016; Schopf *et al.* 2017; Avellaneda *et al.* 2017). In addition, HSP90 plays an important role in directing misfolded proteins for proteasomal degradation (Taipale *et al.* 2014; Labbadia and Morimoto 2015; Schopf *et al.* 2017). Although HSP90 is absent from Archea, it is found throughout Eubacteria and Eukarya and is an essential protein in numerous eukaryotes, including *S. cerevisiae*, *C. elegans*, *D. melanogaster*, and vertebrates (Borkovich *et al.* 1989; Rutherford and Lindquist 1998; Lele *et al.* 1999; Voss *et al.* 2000; Birnby *et al.* 2000).

HSP90 has been implicated in numerous human diseases, including neurodegenerative diseases and cancer. With respect to neurodegeneration, several disorders, including Alzheimer’s Disease, Parkinson’s Disease, and Huntington’s Disease, involve misfolding and aggregation of proteins, perhaps as a result of HSP90 dysfunction (Pratt *et al.* 2015; Lackie *et al.* 2017). In many types of cancer, HSP90 and other components of the molecular chaperone network are overexpressed, enabling the maturation of many mutant proliferative signaling kinases and transcription factors (*i.e.*, oncoproteins), thereby contributing to the growth factor independent growth and unregulated proliferation that are two of the hallmarks of cancer (Jarosz 2016; Calderwood and Gong 2016; Wu *et al.* 2017). In their wild-type state, these regulatory proteins are also HSP90 clients, pointing to a key role for HSP90 in control of normal cellular proliferation in growth and development (Rutherford *et al.* 2007a, 2007b; Schopf *et al.* 2017).

The Notch pathway, an important signaling pathway, is also disrupted in a variety of genetic diseases and cancers in humans (Aster *et al.* 2017; Mašek and Andersson 2017; Siebel and Lendahl 2017). Notch has arguably been best-studied in the context of development in *D. melanogaster* and *C. elegans* (Greenwald and Kovall 2013; Kimble and Seidel 2014; Kovall *et al.* 2017; Siebel and Lendahl 2017). In *C. elegans*, germline proliferation requires inductive signaling from the somatic gonad to the germline mediated by GLP-1, one of two *C. elegans* Notch orthologs along with LIN-12 (Greenwald and Kovall 2013). Critical components of this signaling pathway include: DSL-type ligands, LAG-2 and APX-1; GLP-1/Notch receptor; and downstream transcriptional regulators, LAG-1 and SEL-8/LAG-3 (Hansen and Schedl 2013; Kimble and Seidel 2014). Upon ligand-receptor binding, a pair of proteolytic cleavages releases the Notch intracellular domain (NICD) for transport to the nucleus where it nucleates formation of a transcriptional activator complex (Greenwald and Kovall 2013). When GLP-1/Notch signaling is absent or reduced in the *C. elegans* gonad, germline stem cells prematurely exit mitosis, enter meiosis, and form gametes (Hansen and Schedl 2013; Greenwald and Kovall 2013; Kimble and Seidel 2014).

Genetic studies in *C. elegans* have identified many of the core components of the Notch signaling pathway and numerous regulators of Notch signaling (Greenwald and Kovall 2013). Interestingly, several of these regulators are predicted to be components of the proteostasis network, including proteasome subunit PAS-5 and ubiquitin E3 ligases UBR-5, SEL-10, and RFP-1 (Hubbard *et al.* 1997; MacDonald *et al.* 2008; Gupta *et al.* 2015; Safdar *et al.* 2016). One fruitful approach for identifying Notch pathway regulators has been to use a sensitized genetic background, temperature-sensitive *glp-1(bn18)* mutants raised at semi-permissive temperature, to recover genetic enhancers of the *glp-1* reduced germline proliferation phenotype (*ego* mutants) (Qiao *et al.* 1995; Maine *et al.* 2004; Liu and Maine 2007; She *et al.* 2009). This approach has identified factors that promote germline proliferation, either as components or regulators of GLP-1/Notch signaling or in parallel with GLP-1/Notch signaling.

Here we describe the molecular identification of another positive regulator of germline proliferation identified by this approach, *ego-3*. The single *ego-3* allele recovered in initial screens, *ego-3(om40)*, has a pleiotropic phenotype suggesting the *ego-3* gene product is active in numerous tissues throughout the course of development (Qiao *et al.* 1995). We report here that *ego-3* encodes the *C. elegans* ortholog of the cytosolic form of the HSP90 molecular chaperone. Our molecular, genetic, and phenotypic analyses show that HSP-90 is required for GLP-1/Notch signaling in *C. elegans* germline stem cells and provide the first evidence that HSP90 plays an essential role in Notch signaling in development.

## Materials and Methods

### Nematode strains and culture

Worms were maintained on *E. coli* strain OP50 seeded on NGM-Lite agar plates (Sun and Lambie 1997) under standard conditions (Epstein and Shakes 1995). Wild-type strains used were *C. elegans* variant Bristol (N2) and CB4856 (Hawaiian strain). Mutant strains used in this study were: BE63 *sqt-3(sc63)*, CB1489 *him-8(e1489)*, DG2389 *glp-1(bn18)*, DR96 *unc-76(e911)*, EL44 *unc-32(e189) glp-1(bn18)*, EL129 *hsp-90(om40) unc-76(e911)/nT1[unc-?(n754) let-?]*, EL160 *lag-1(om13ts)/nT1[unc-?(n754) let-?]*; *hsp-90(om40)/nT1[unc-?(n754) let-?]*, EL301 *lag-1(om13ts)*, EL375 *sqt-3(sc63) unc-76(e911)*, EL384 *sqt-3(sc63) hsp-90(om118) unc-76(e911)/sdc-3(y52y180) unc-76*, EL418 *hsp-90(om118) unc-76(e911)/nT1[unc-?(n754) let-?]*, EL490 *unc-39(e257) hsp-90(om40)/unc-39(e257) unc-76(e911)*, EL660 *lag-1(om13ts)/mIs11him-8(e1489)*, JL48 *unc-32(e189) glp-1(bn18)*; *hsp-90(om40)/nT1[qIs51]*, JL49 *hsp-90(om40)/nT1[qIs51]*, JL54 *unc-32(e189) glp-1(bn18)*; *hsp-90(p673)/nT1[qIs51]*, JT6130 *hsp-90(p673)*, TY1470 *yDf8/nT1[unc-?(n754) let-?]*, VC914 *hsp-90(ok1333)/nT1[qIs51]*, YY216 *eri-9(gg106)*. Note: The *daf-21* gene name was recently changed to *hsp-90*. Accordingly, the strains listed here are referred to using *hsp-90* rather than *daf-21* and *ego-3*.

### RNAi feeding assay

RNAi of *hsp-90* was performed by the feeding method (Timmons *et al.* 2001). A 2258 bp genomic region containing nearly the entire *hsp-90* protein-coding region was amplified by polymerase chain reaction from wild-type N2 genomic DNA using 5′-TGTCCGAGAACGCCGAAA-3′ and 5′-GTCGACCTCCTCCATGCG-3′ as primers. The resulting amplicon was cloned into RNAi feeding vector L4440 and then transformed into *E. coli* RNAi feeding strain HT115 (Timmons *et al.* 2001). RNAi was performed by placing animals of a particular strain and stage onto *hsp-90* feeding plates at a specified temperature as described in the Results. Adults were examined by differential interference contrast microscopy or were stained with DAPI to visualize DNA and examined by fluorescence microscopy (see below). An *E*. *coli* feeding strain expressing GFP dsRNA was used as a negative control.

### Isolation of om118 by non-complementation screen

*om40* non-complementation screens were performed using either UV or EMS mutagenesis in an effort to isolate additional alleles of *ego-3*. UV mutagenesis was performed as described (Yandell *et al.* 1994) and yielded a single mutation, *om118*, that failed to complement the *om40* germline and somatic phenotypes. In brief, *sqt-3(sc63) unc-76(e911)* hermaphrodites were treated with 30 ug/ml trimethylpsoralen (TMP) in M9 buffer for 15 min in the dark and irradiated with a long-wave UV source (Model UVGL-25, UVP, Inc.) at distance of 10 cm for 20 sec. The *sqt*-3*(sc63)* mutation confers recessive squat (Sqt) and dominant roller (Rol) phenotypes. Single candidate *sqt-3(sc63) ego-3(omx) unc-76(e911)/sqt-3(sc63) ego-3(+) unc-76(e911)* F1 hermaphrodites were mated with *ego-3(om40) unc-76(e911)/++* males. Rol Unc cross-progeny (*sqt-3(sc63) ego-3(omx) unc-76(e911)/sqt-3(+) ego-3(om40) unc-76(e911)* or *sqt-3(sc63) ego-3(+) unc-76(e911)/sqt-3(+) ego-3(om40) unc-76(e911)*) were screened for the Ego-3 sterile phenotype, which would presumably be of the genotype *sqt-3(sc63) ego-3(omx) unc-76(e911)/+ ego-3(om40) unc-76(e911)*. The *om118* mutation was identified from a total of 30,000 haploid genomes and recovered from fertile *sqt-3(sc63) ego-3(om118) unc-76(e911)/*+++ Rol non-Unc siblings.

### Single nucleotide polymorphism (SNP) mapping

We mapped *ego-3(om40)* relative to SNPs as described (Maine *et al.* 2004). We put *unc-39(e257) ego-3(om40)* and *ego-3(om40) unc-76(e911)* chromosomes, generated in the N2 strain background, over chromosomes from the polymorphic wild-type strain, CB4856, and picked *unc-39(e257)* non-*ego-3(om40)* and *unc-76(e911)* non-*ego-3(om40)* recombinants, respectively. Using data available in WormBase (www.wormbase.org), we identified candidate SNPs in the region and verified their identity by PCR amplification and Sanger sequencing. We isolated homozygous recombinants from each Unc non-Ego recombinant line, PCR amplified the SNP region, and assayed by differential restriction digest or Sanger sequencing, as appropriate. This approach mapped *ego-3(om40)* to the right of two SNPs in the region covered by cosmid C54G10 and to the left of three SNPs in the region covered by Y50E8A, placing it within a 70 kb interval.

### Whole genome sequencing (WGS)

#### Growth and isolation of ego-3(om40) homozygotes*:*

Strain JL49, *ego-3(om40)/nT1[qIs51]*, contains *ego-3(om40)* balanced by a variant of the reciprocal translocation *nT1* marked with an integrated *myo-2*::GFP construct that confers pharyngeal GFP expression. JL49 exhibits pharyngeal GFP and segregates sterile non-GFP *ego-3(om40)* homozygotes and GFP heterozygotes as the live progeny. The homozygous balancer is recessive lethal, and the strain also segregates a large percentage of nonviable aneuploid progeny.

JL49 was harvested from a starved 60 mm plate using M9 buffer supplemented with 0.01% Triton X-100, pelleted by centrifugation, and replated on 100 mm rich agarose plates (standard NGM recipe with 8X peptone and substituting low-EEO agarose at 1:1 for agar) seeded with lawns of *E. coli* strain OP50 or χ1666. Populations were allowed to grow at 20° until dense with growing, well-fed worms of mixed stage, then harvested by M9/Triton X-100 wash and centrifugation. Excess bacterial food was removed by three to five further rounds of washing and pelleting.

The resulting population was flow-sorted using a Copas Biosort 250 with fluorescence detection and Biosorter software version 5.25.2. A sorter screening window was chosen to select worms at the L4 stage or older. This set was further restricted to animals not expressing pharyngeal GFP, using the fluorescence-detection capability of the Copas sorter, resulting in a very highly enriched sample of *ego-3(om40)* homozygotes for whole genome sequencing.

The sorted homozygotes were replated on standard 60 mm NGM plates, and any remaining GFP animals were removed by hand picking. The contamination rate for GFP animals was very low, approximately 40 GFP animals in 15,000 total sorted worms.

#### Library preparation and sequencing*:*

The sample of *ego-3(om40)* homozygotes was frozen at -80°. Genomic DNA was prepared using a standard protocol of Proteinase K digestion, treatment with RNAse A and 6 M NaCl, precipitation in isopropanol, washing, and resuspension in distilled water. A sequencing library was generated using the Nextera XT library preparation protocol, and the resulting library was sequenced on an Illumina HiSeq machine in the laboratory of Dr. Corey Nislow (Department of Pharmaceutical Sciences, University of British Columbia). Paired-end reads of 100 bp were generated, resulting in total genome coverage of 57X.

#### Sequence analysis*:*

Single-nucleotide variants (SNVs) were called with the analysis pipeline and filters developed for the Million Mutation Project (Thompson *et al.* 2013). Briefly, sequence read pairs were aligned to the *C. elegans* reference genome version WS230 (www.wormbase.org) using the short-read aligner Phaster (P. Green, personal communication). SNVs were then identified with SAMtools (Li *et al.* 2009) and annotated with a custom Perl script.

### Sanger DNA sequencing

The *om40* molecular lesion identified by WGS was confirmed by PCR amplification of the region containing the putative mutation from *om40* homozygotes followed by Sanger sequencing. The *om118* molecular lesion was identified by amplifying each predicted *hsp-90* exon, including exon-intron junctions, by PCR from *ego-3(om118) unc-76(e911)/nT1[unc-?(n754) let-?]* heterozygotes followed by Sanger DNA sequencing. Sequence data suggested the presence of an insertion mutation in *hsp-90*. To determine unambiguously the location and sequence of the insertion, the region containing the putative insertion was again amplified from *ego-3(om118) unc-76(e911)/nT1[unc-?(n754) let-?]* heterozygotes, and PCR products were cloned into pUC19 using standard techniques. Plasmid DNA was isolated from 12 independent clones and sequenced. All clones contained either wild-type sequence or the *om118* insertion mutation. PCR primer sequences and their locations can be found in Table S3 and Figure S1 in File S1, respectively.

### Microscopy and DAPI methods

Microscopy was carried out with a Zeiss Axioskop equipped with differential interference contrast (DIC) optics and epifluorescence. For fluorescence visualization of nuclei, intact worms were fixed in -20° methanol, and dissected gonads were fixed with 3% paraformaldehyde. Fixed tissue was incubated for 10-15 min in 0.2 ug/ml DAPI at room temperature and mounted in Vectashield (Vector Laboratories).

### Data availability

All *C. elegans* strains and plasmid constructs used in this study are available upon request. The raw sequence data from the WGS have been submitted to the NCBI BioProject (http://www.ncbi.nlm.nih.gov/bioproject) under accession number PRJNA428227 and can be accessed from the Sequence Read Archive (SRA; https://www.ncbi.nlm.nih.gov/sra) with accession number SRP127800. File S1 contains Figure S1, Locations of primers used to amplify and sequence regions of the *hsp-90* gene to identify mutations; Figures S2–S4 in File S1, Sanger DNA sequencing chromatograms of wild-type and mutant sequences; Table S1 in File S1, Gonad transcriptome data for protein-coding genes in the *ego-3* region; Table S2 in File S1, Codon and splicing changes detected in *ego-3(om40)* by whole genome sequence analysis; and Table S3 in File S1, Primers used to amplify and sequence regions of the *hsp-90* gene to identify mutations.

## Results

One goal of the *glp-1* enhancer screen was to isolate partial loss-of-function alleles of genes that promote GLP-1/Notch signaling (Qiao *et al.* 1995). The rationale was to recover mutations that cause premature loss of all germline stem cells in a sensitized genetic background where GLP-1 activity is suboptimal, but do not cause the loss of germline stem cells when GLP-1 activity is normal. For example, *glp-1(bn18ts)*; *ego-3(om40)* adults do not maintain a population of proliferative germ cells, but instead all germ cells enter meiosis prematurely and undergo gametogenesis (Qiao *et al.* 1995). In a *glp-1(+)* background, in contrast, *ego-3(om40)* adults maintain a proliferative germ line. During development, *ego-3(om40)* alone also causes a number of defects in somatic and germline tissues including delayed development, paralyzed movement (an Unc phenotype), and “early” and “late” germline defects (Qiao *et al.* 1995). Notably, the Unc phenotype is much less severe in adults than in larvae. The early germline phenotype is a transient pause in larval germline proliferation from early L3 to early/mid-L4 stages; the late germline phenotype includes delayed onset of meiosis and gametogenesis, sterility, and development of a proximal germ cell tumor. Most aspects of the *ego-3(om40)* phenotype appear to be loss-of-function based on analysis of *ego-3(om40)/yDf8* individuals (Qiao *et al.* 1995). However, the proximal tumor phenotype is milder in *ego-3(om40)/yDf8* animals, suggesting this aspect of the phenotype may not be due to a strict loss-of-function.

### Isolation of a strong ego-3 loss-of-function allele

To identify additional alleles of *ego-3*, we carried out a non-complementation screen using UV/trimethylpsoralen (TMP) mutagenesis. One mutation, *ego-3(om118)*, was identified that fails to complement *ego-3(om40)* for the germline and somatic developmental defects described above and for *glp-1* enhancement. In contrast, *ego-3(om118)* homozygotes and *ego-3(om118)/yDf8* transheterozygotes die as embryos, thus *ego-3(om118)* appears to be a more severe loss-of-function allele than *ego-3(om40)*. Consistent with this hypothesis, the *ego-3(om118/om40)* Unc phenotype is more severe than for *ego-3(om40)* animals, persisting into adulthood rather than being limited to larval stages.

### ego-3 is allelic to daf-21 and encodes HSP90

*ego-3(om40)* maps just to the left of *unc-61* on LGV (Qiao *et al.* 1995). To determine the molecular identity of *ego-3*, we performed additional three-factor and SNP mapping to refine the location of *ego-3*. Three-factor mapping placed *ego-3(om40)* to the right of or very close to the left of *daf-21*, which was recently renamed *hsp-90* (www.wormbase.org). Specifically, 23/23 Unc non-Ego recombinants from an *hsp-90(p673)/ego-3(om40) unc-76(e911)* strain were *hsp-90(p673) unc-76(e911)/ego-3(om40) unc-76(e911)*. Note that *hsp-90(p673)* is a recessive gain-of-function mutation causing a dauer-constitutive (Daf-c) phenotype (Birnby *et al.* 2000), and therefore would be expected to complement a loss-of-function mutation in the same gene.

**Figure 1:**
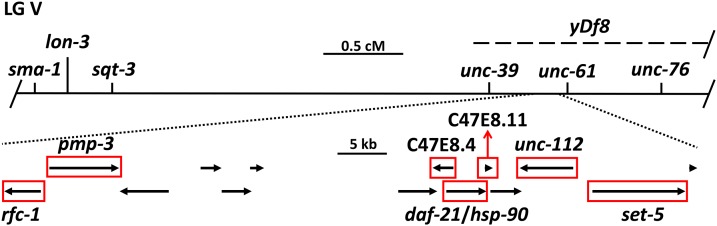
Genetic and physical maps of *ego-3* region. The genetic map of the *ego-3* region is shown at the top along with the location of the *yDf8* deletion used in this study. SNP mapping localized *ego-3* to a region to the left of *unc-61*. The physical map of this region, obtained from WormBase WS259 and showing only protein-coding genes, is shown below the genetic map. Arrows indicate location, length, and orientation of genes. Red boxes indicate genes with substantial expression in the gonad (Table S1 in File S1).

We next mapped *ego-3(om40)* relative to SNPs in the *unc-39* to *unc-76* interval, further localizing it to an ∼70 kb region containing 14 predicted protein-coding genes and several non-coding RNA genes (Figure 1). Gonad transcriptome data (Guo *et al.* 2015) indicated that nine of these genes are expressed in the gonad (Table S1 in File S1). As none was an obvious candidate for involvement in GLP-1/Notch signaling, we undertook to identify the *ego-3(om40)* mutation by whole genome sequencing (WGS).

**Figure 2:**
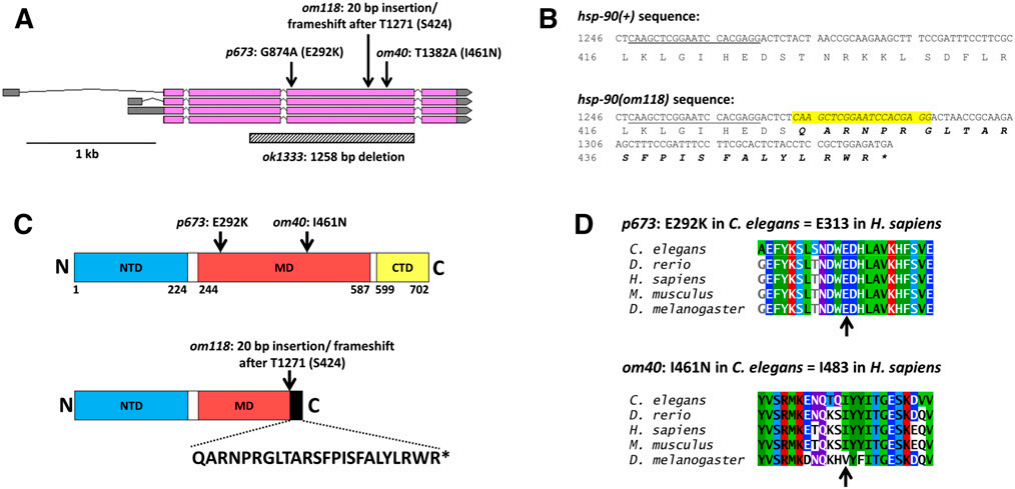
Molecular analysis of *hsp-90* alleles. A. *hsp-90* is predicted to encode four transcript isoforms, all of which have the same predicted amino acid sequence. Arrows indicate locations of new and previously identified *hsp-90* alleles. The locations of *p673*, *om118*, and *om40* were determined by Sanger sequencing, and the location of *ok1333* was obtained from WormBase. The nucleotide positions of the mutations are numbered from the first nucleotide of the protein-coding sequence; numbering does not include nucleotides in introns. Amino acid positions in parentheses are numbered from the initiator methionine. Numbering is from WormBase WS259. B. Partial DNA and amino acid sequences of *hsp-90(+)* and *hsp-90(om118)* insertion/frameshift mutation. Numbers indicate nucleotide positions in the protein-coding sequence and amino acid positions in the predicted translation product. The underlined nucleotide sequence is duplicated and inserted 5 bp downstream in *hsp-90(om118)*; the inserted sequence is highlighted in yellow. Amino acids changed as a result of the insertion are shown in bold italics. *Premature termination codon. C. Schematic diagram of *C. elegans* HSP-90 domain organization and location of mutations. Top, schematic diagram showing locations of *p673* and *om40* missense mutations. Bottom, schematic diagram showing location of *om118* insertion mutation. The black box indicates extent of the amino acid sequence changes caused by the insertion and resulting frameshift. The sequence of amino acids added to HSP-90 after S424 are shown. The predicted length of the mutant protein is 448 amino acids. Amino acid sequences of *C. elegans* HSP-90 (NP_506626.1) and *H. sapiens* HSP90AA1 (NP_005339.3) were aligned using NCBI Protein BLAST (blastp) to identify corresponding amino acid residues and HSP90AA1 domain boundaries from Haase and Fitze 2016 were used to identify the corresponding boundaries in HSP-90. Numbers indicate amino acid residues in *C. elegans* HSP-90. NTD, N-terminal domain; MD, middle domain; CTD, C-terminal domain. D. Partial amino acid sequence alignment of HSP90 from *C. elegans* and selected metazoans. Top, alignment of region containing the *p673* mutation. Bottom, alignment of region containing the *om40* mutation. The corresponding amino acid number in *H. sapiens* for each mutation is shown. Arrow, location of amino acid affected by the indicated mutations.

WGS of *ego-3(om40)* homozygotes identified one sequence variant within the protein-coding region of one gene in the mapped region, *hsp-90*, which encodes the *C. elegans* ortholog of the cytoplasmic form of the molecular chaperone HSP90 (Table S2 in File S1) (Birnby *et al.* 2000). The *om40* sequence variant is a T→A transition causing an I→N non-conservative missense mutation (Figure 2A), which we confirmed by Sanger sequencing (Figure S2 in File S1). Subsequently, we amplified and Sanger sequenced all *hsp-90* exons from *ego-3(om118)* to locate the *om118* lesion. We discovered a 20 bp non-tandem duplication within the same exon containing the *om40* mutation, which is predicted to cause a frameshift and to introduce a premature termination codon (Figure 2B and Figure S4 in File S1). The inserted sequence occurs only once in the *C. elegans* wild-type genome and is therefore highly likely to have originated from within the *hsp-90* gene. These data are strong evidence that *hsp-90* and *ego-3* are the same gene.

To confirm that *ego-3* and *hsp-90* are indeed the same gene, we tested for complementation between *ego-3(om40)* and *hsp-90(ok1333)*, a deletion and presumed null allele (Figure 2A) (*C. elegans* Deletion Mutant Consortium 2012). *hsp-90(ok1333)* worms have a paralyzed Unc phenotype and arrest development during mid to late larval stages (www.wormbase.org). We crossed *ego-3(om40)/nT1[qIs51]* males with *hsp-90(ok1333)/nT1[qIs51]* hermaphrodites at 20°. The *nT1[qIs51]* balancer chromosome has pharyngeal GFP expression, so non-GFP progeny will be *hsp-90(ok1333)* homozygous self-progeny and *hsp-90(ok1333)*/*ego-3(om40)* cross-progeny. Large numbers of GFP males, presumably *ego-3(om40)/nT1[qIs51]* cross-progeny, were produced, indicating that the *ego-3(om40)/nT1[qIs51]* males mated efficiently.

The vast majority of non-GFP offspring from the cross were paralyzed Uncs and arrested as larvae, as expected if *hsp-90(ok1333)* and *ego-3(om40)* do not complement. A few non-GFP worms survived to adulthood, with most developing into sterile, severe Unc hermaphrodites and severe Unc males. Therefore, *ego-3(om40)* fails to complement *hsp-90(ok1333)*, and *ego-3* is allelic to *hsp-90*, consistent with our molecular analysis of *ego-3* alleles.

Interestingly, the *ego-3(om40)* larval phenotype resembles the *daf-21(ok1333)* phenotype in that animals are paralyzed, develop slowly, and some aspects of development arrest during L2-L3 stage (www.wormbase.org; Qiao *et al.* 1995). However, both somatic and germline development arrest in *hsp-90(ok1333)* mutants whereas only germline development arrests in *ego-3(om40)*. We interpret the viability and complementation of *om40/p673* transheterozygotes (constructed during genetic mapping, see Materials and Methods) as resulting from the previously reported gain-of-function nature of the *p673* allele (Birnby *et al.* 2000).

Additionally, we determined the nucleotide sequence change in *hsp-90(p673)*, which had not been previously reported although the amino acid sequence change for this allele had been (Birnby *et al.* 2000). We found a G→A transition mutation at the expected location, confirming the presence of the previously identified non-conservative E→K missense mutation (Figure 2A and Figure S3 in File S1).

The *om40*, *om118*, and *p673* alleles are all predicted to cause amino acid sequence changes affecting the middle domain (MD) of HSP90 (Figure 2C). Both the *p673* and *om40* alleles are non-conservative missense mutations of highly conserved residues in metazoans (Figure 2D) (Birnby *et al.* 2000). Furthermore, *om118* is predicted to lead to loss of much of the middle domain and all of the carboxy-terminal domain (CTD) of HSP90. The MD is essential for interactions with both client proteins and co-chaperones, and it contains a catalytic loop that contributes to ATP hydrolysis (Röhl *et al.* 2013; Haase and Fitze 2016; Schopf *et al.* 2017). The CTD is the dimerization domain of HSP90, and it contains a highly conserved tetratricopeptide repeat (TPR) domain binding site required for interactions with many co-chaperones (Röhl *et al.* 2013; Haase and Fitze 2016; Schopf *et al.* 2017).

Based on our molecular and genetic data, we conclude that *ego-3* and *hsp-90* are the same gene. In keeping with chaperone nomenclature in other organisms, the *ego-3* and *daf-21* gene names have been changed to *hsp-90* (www.wormbase.org).

### hsp-90(p673) does not enhance glp-1(bn18)

**Figure 3:**
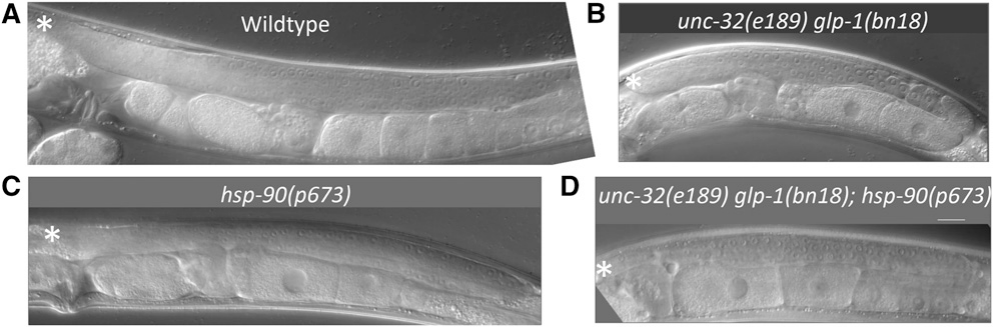
*hsp-90(p673)* does not enhance the germline proliferation defect in *glp-1(bn18)*. Germline morphology of animals raised at 20°. Adult hermaphrodites were photographed at the same magnification with DIC microscopy; one gonad arm from representative animals is shown in each panel. Asterisk indicates the distal end of the germline. A. N2 (wild-type) adult hermaphrodite gonad has a large germline with extensive proliferation. B. *unc-32(e189) glp-1(bn18ts)* hermaphrodites are fertile but have approximately half as many germ cells as wild type (N2) (Qiao *et al.* 1995). *unc-32(e189)* is a visible genetic marker that does not affect germline development. C. *hsp-90(p673)* hermaphrodites are fertile with a wild-type germline morphology. D. *unc-32(e189) glp-1(bn18ts)*; *hsp-90(p673)* hermaphrodites are fertile and have a germline similar in size to the *unc-32(e189) glp-1(bn18ts)* germline.

We hypothesized that *hsp-90(p673)* should not enhance *glp-1(bn18)*, because *hsp-90(p673)* is a gain-of-function allele (Birnby *et al.* 2000), and it also complements *hsp-90(om40)* (see above). To test this, we constructed an *unc-32(e189) glp-1(bn18)*; *hsp-90(p673)/nT1[qIs51]* strain. *unc-32(e189) glp-1(bn18)*; *hsp-90(p673)* offspring are viable and fertile at 20° and produce a large proportion of dauer offspring, as expected for *hsp-90(p673)* animals. We also used DIC microscopy to examine the germlines of *unc-32(e189) glp-1(bn18)*; *hsp-90(p673)* worms raised at 20° to see if *hsp-90(p673)* enhanced the mild germline proliferation defect in *glp-1(bn18)*. *hsp-90(p673)* germlines resemble N2 wild-type germlines with extensive germline proliferation (Figure 3, A and C). In contrast, *unc-32(e189) glp-1(bn18)* worms raised at 20° have substantially smaller germlines because of reduced GLP-1/Notch signaling (Figure 3B) (Qiao *et al.* 1995). *unc-32(e189) glp-1(bn18)*; *hsp-90(p673)* germlines closely resemble those of *unc-32(e189) glp-1(bn18)* animals (Figure 3D), therefore *hsp-90(p673)* does not enhance *glp-1(bn18)*. Taken together, our results show that the *hsp-90(om40)* and *hsp-90(p673)* alleles both retain substantial HSP-90 activity such that the heteroallelic combination has approximately normal HSP-90 function. We further conclude that only a subset of *hsp-90* mutations have the ability to enhance *glp-1*.

### The glp-1(bn18) phenotype is enhanced by the loss of HSP90 activity

Our previous study determined that the *glp-1(bn18)* germline proliferation defect is enhanced in *hsp-90(om40)/yDf8*; *glp-1(bn18)* animals (Qiao *et al.* 1995), strongly suggesting a loss of HSP-90 activity is responsible for the enhancement. In addition, the early germline (arrested proliferation) and uncoordinated phenotypes were similar in *hsp-90(om40)/yDf8*; *glp-1(+)* and *hsp-90(om40/om40)* mutants, consistent with those defects arising from reduced HSP-90 activity. However, the late germline phenotype, including proximal germline proliferation (Pro phenotype) and delayed gametogenesis, was less severe in *hsp-90(om40)/yDf8* than in *hsp-90(om40/om40)* adults. At the time, we hypothesized this result might indicate that *hsp-90(om40)* has some gain-of-function character.

Molecular identification of the *ego-3* gene has now allowed us to investigate the nature of the *om40* allele in more detail. The lethal *hsp-90(ok1333)* allele appears to be null (as described above) and the *yDf8* deletion completely uncovers the *hsp-90* gene; therefore, the viability of *hsp-90(om40/ok1333)* and *hsp-90(om40)/yDf8* confirm that *hsp-90(om40)* is not a null allele. At the same time, similarities among *hsp-90(om40)*, *hsp-90(om40/ok1333)*, and *hsp-90(om40)/yDf8* phenotypes suggest that *hsp-90(om40)* predominantly causes a loss of gene function. To confirm this interpretation and to investigate the nature of the proximal proliferation defect, we knocked down HSP-90 via RNAi and examined the impact on development. Our first strategy was to place L4 wild-type (N2) hermaphrodites onto feeding plates containing bacteria expressing *hsp-90* dsRNA at 20° and evaluate their F1 progeny. This strategy partially phenocopied *hsp-90(om40)*, yielding offspring that were sterile, many with very reduced germline proliferation. Knockdown also caused some embryonic lethality in the F1, a phenotype observed in *hsp-90(ok1333)* and *hsp-90(om118)*, and a protruding vulva phenotype. We observed similar results in *unc-32(e189) glp-1(bn18)*; *hsp-90(RNAi)* animals with the additional observation of some embryonic lethality. We also performed *hsp-90(RNAi)* in an enhanced RNAi mutant, *eri-9(gg106)*, and saw the same phenotypes, with the addition that some larvae arrested development. We next placed L1 larvae onto *hsp-90* RNAi plates at 20° and examined the adults three days later. In a wild-type background, *hsp-90(RNAi)* adults were sterile with protruding vulvas and had very reduced germlines; the same results were obtained for *unc-32(e189) glp-1(bn18)*; *hsp-90(RNAi)* although a smaller proportion of animals had a protruding vulva. *eri-9(gg106) hsp-90(RNAi)* adults were also sterile, but most animals were Unc and very sick with several having died in early adulthood. In no case did we observe the proximal germline proliferation (Pro) characteristic of *hsp-90(om40)*.

**Figure 4:**
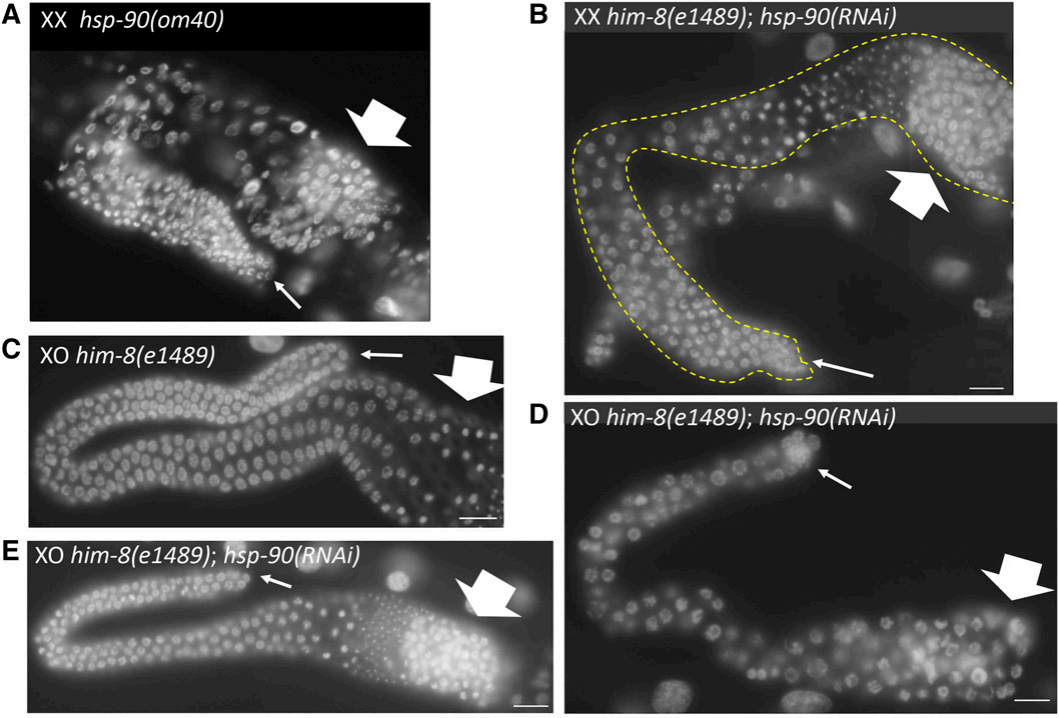
*hsp-90(RNAi)* phenocopies *hsp-90(om40)* germline phenotype. Germline nuclei from adult worms were stained with DAPI to reveal chromosome morphology. Thick arrows and thin arrows indicated proximal and distal regions of the germline, respectively. Scale bar, 16 um. A. Gonad from intact *hsp-90(om40)* adult hermaphrodite (XX) showing the Pro phenotype characteristic of *hsp-90(om40)*. Mitotic nuclei are visible in both distal and proximal regions. B. Gonad dissected from a *him-8(e1489)*; *hsp-90(RNAi)* adult hermaphrodites (XX). Gonad outlined with yellow dashed line shows the Pro phenotype. C. Gonad dissected from a *him-8(e1489)* adult male (XO) displays wild-type germline nuclear morphology. Mitotic nuclei are visible in the distal region and sperm (smallest bright spots) are present in the proximal region. D. Gonad dissected from a *him-8(e1489)*; *hsp-90(RNAi)* adult male (XO) showing large nuclei in the distal region. E. Gonad dissected from a *him-8(e1489)*; *hsp-90(RNAi)* adult male (XO) showing the Pro phenotype.

As a follow up experiment, we assayed the effect of beginning *hsp-90* RNAi at L3 stage, reasoning that allowing some germline development to occur prior to knocking down HSP-90 might increase the chance of observing the Pro phenotype observed in *hsp-90(om40)* adults (Figure 4A). We performed this study with a *him-8(e1489)* strain in order to evaluate both males and hermaphrodites. Untreated *him-8(e1489)* worms have normal germlines (Figure 4C). The majority of *hsp-90(RNAi*) adults produced in this study had germline developmental defects, often resembling either the *hsp-90(om40)* early or late germline defects (Figure 4, B, D, and E). Approximately 73% of germlines were small and germ cell nuclei had an enlarged morphology similar to the *hsp-90(om40)* early germline phenotype (n = 52; Figure 4D); this *hsp-90(RNAi)* phenotype is consistent with previous observations (Waters *et al.* 2010). The remaining ∼27% of germlines had more extensive proliferation and produced gametes. A subset of these, ∼6% of germlines assayed, had proximal proliferation (Figure 4, B and E) resembling the *hsp-90(om40)* Pro phenotype (Figure 4A). Therefore, we conclude that the *hsp-90(om40)* Pro phenotype results from reduced HSP90 activity.

### Loss of HSP-90 activity also enhances lag-1(om13ts)

To further explore the role of HSP-90 in promoting Notch signaling, we asked whether *hsp-90(om40)* enhances the loss of *lag-1* function in the germline. LAG-1 is a CSL-type transcription factor active downstream of GLP-1 in the *C. elegans* germline (Hansen and Schedl 2013; Kimble and Seidel 2014). Here, we used a temperature sensitive allele, *lag-1(om13ts)*, that is fertile at 20° (Qiao *et al.* 1995; Safdar *et al.* 2016). We reasoned that the combination of partial loss-of-function mutations in two genes involved in GLP-1/Notch signaling should substantially reduce germline proliferation. We generated a *lag-1(om13ts)/nT1[unc-?(n754) let-?]*; *hsp-90(om40)/nT1[unc-?(n754) let-?]* strain and evaluated germline proliferation in the *hsp-90(om40)*; *lag-1(om13ts)* progeny raised at 20°. *hsp-90(om40)* and *lag-1(om13ts)* single mutants, each obtained from balanced strains, were assayed in parallel; all animals were stained with DAPI as ∼1 day old adults. As expected based on our previously published data, germline proliferation was retained in 100% of *hsp-90(om40)* and *lag-1(om13ts)* single mutants (Table 1) (Qiao *et al.* 1995; Safdar *et al.* 2016). In contrast, 100% of *lag-1(om13ts)*; *hsp-90(om40)* adult hermaphrodite germlines contained sperm, but lacked proliferating and meiotic germ cells (Table 1). Hence, in these double mutants, all proliferating germ cells prematurely exit mitosis, enter meiosis, and undergo gametogenesis. The germ cell precursors in these *lag-1(om13ts)*; *hsp-90(om40)* animals apparently divided only a few times, as an average of only 26 sperm were observed per gonad arm (range 10-46; n = 12), corresponding to an average of 6-7 germ cells. This result is consistent with a severe reduction in GLP-1 signaling in the *lag-1(om13)*; *hsp-90(om40)* double mutant and further supports our conclusion that HSP-90 activity promotes GLP-1 pathway signaling activity.

**Table 1:** *hsp-90(om40)* enhances *lag-1(om13ts)* in the germline

Genotype	N*^a^*	% with distal mitotic GC*^b^*	% with meiotic GC*^b^*	% with gametes (type)
*lag-1(om13)*; *hsp-90(om40)*	62	0	0	100 (sperm)
*lag-1(om13ts)*	30	100	100	100 (sperm + oocytes)
*hsp-90(om40)*	32	100	94	78 (sperm +/or oocytes)*^c^*

a N, number of gonad arms assayed.

b GC, germ cells.

c The phenotype of ∼1 day old adult *hsp-90(om40)* hermaphrodites was variable with respect to gamete production and presence of a proximal tumor, as previously described (Qiao *et al.* 1995).

Assays were performed at 20°. Mutations were maintained as balanced heterozygotes. Homozygous mutant offspring were identified and assayed within the first 24 hr of adulthood.

## discussion

By various genetic and molecular criteria, we have determined the identity of *ego-3* as *hsp-90*, which encodes the *C. elegans* ortholog of the molecular chaperone HSP90. Our findings that *hsp-90(om40)* enhances mutations in both the GLP-1/Notch receptor and the LAG-1 downstream effector demonstrate that HSP-90 activity directly or indirectly promotes GLP-1/Notch signaling and is therefore required for proper germline stem cell function. While HSP90 has long been known to participate in a variety of developmental as well as proliferative/oncogenic signaling pathways in metazoans (Whitesell and Lindquist 2005; Rutherford *et al.* 2007b; Green *et al.* 2011; Haslbeck *et al.* 2012; Garg *et al.* 2016; Calderwood and Gong 2016; Wu *et al.* 2017; Chatterjee and Burns 2017), our results provide the first evidence that HSP90 plays a role in Notch signaling in development. Recently, direct physical interaction between HSP90 and Notch intracellular domain (NICD) has been demonstrated in cultured human cells (Deskin *et al.* 2016; Wang *et al.* 2017), consistent with our results and with a direct role for HSP90 in Notch signaling.

Our discovery that HSP-90 modulates GLP-1/Notch signaling in *C. elegans* adds to the number of proteostasis network components known to regulate Notch function in various organisms. These components are also important in stem cell function in general (Lee *et al.* 2017; Noormohammadi *et al.* 2017; Werner *et al.* 2017) and in *C. elegans* germline stem cell function in particular. In addition to direct interaction between NICD and HSP90, Notch signaling is altered by loss of or reduced function of ubiquitin E3 ligases and the proteasome (MacDonald *et al.* 2008; Gupta *et al.* 2015; Safdar *et al.* 2016; Carrieri and Dale 2017; Wang *et al.* 2017; Perez-Mockus and Schweisguth 2017). Our data do not allow us to determine which aspect or aspects of HSP90’s function in proteostasis of GLP-1/Notch, *i.e.*, folding, maturation, interactions with other proteins, or degradation, are necessary for GLP-1/Notch signaling. Systematic investigation of the hundreds of proteins that make up the proteostasis network regulating synthesis, maturation, function, and degradation of the proteome is likely to yield additional factors regulating Notch signaling.

In *C. elegans*, *hsp-90* has previously been shown to be an essential gene that produces a variety of phenotypes when mutated or knocked down by RNAi including sterility, embryonic and larval lethality, constitutive dauer formation, defective motility, and various germline defects (Vowels and Thomas 1994; Piano *et al.* 2000; Rual *et al.* 2004; Gaiser *et al.* 2009, 2011; Waters *et al.* 2010; *C. elegans* Deletion Mutant Consortium 2012; Frumkin *et al.* 2014). These pleiotropic effects are consistent with ubiquitous expression of *hsp-90* in most tissues (except for mature sperm) and throughout the nematode lifecycle from embryonic through adult stages (Yamaguchi *et al.* 1983; Inoue *et al.* 2003; Waters *et al.* 2010; Klosin *et al.* 2017).

The germline phenotypes seen in *hsp-90(om40)* and *hsp-90(RNAi)* animals are consistent with previously observed strong expression of HSP-90 in the developing and mature *C. elegans* germline (Yamaguchi *et al.* 1983; Inoue *et al.* 2003), where the GLP-1/Notch receptor is expressed (Crittenden *et al.* 1994). A germline role for HSP-90 in GLP-1/Notch signaling is also indicated by the observation that the *hsp-90(om40)* larval germline phenotype is epistatic to *glp-1(oz112gf/q224)*, a heteroallelic combination that results in germline overproliferation (Qiao *et al.* 1995; Berry *et al.* 1997). The GLP-1/Notch protein encoded by *glp-1(oz112)* is constitutively active, yet reduction of HSP-90 activity in *glp-1(oz112gf/q224)*; *hsp-90(om40)* larvae is sufficient to reduce GLP-1/Notch signaling and produce the *hsp-90(om40)* phenotype instead. Hence, Qiao *et al.* (1995) concluded that HSP-90 acts downstream of GLP-1/Notch. Furthermore, the recently reported physical interaction between NICD and HSP90 also supports the germline as a location of HSP-90 action in GLP-1/Notch signaling in *C. elegans* (Deskin *et al.* 2016; Wang *et al.* 2017). Of course, none of these observations rules out a separate role for HSP-90 in the signaling cell (*e.g.*, distal tip cell).

The molecular identities of the two known *hsp-90* missense mutations, *hsp-90(p673)* and *hsp-90(om40)*, which cause non-conservative amino acid substitutions of highly conserved residues in the middle domain HSP-90, do not suggest obvious mechanisms by which they might produce their phenotypic effects. *hsp-90(p673)* is a recessive gain-of-function mutation producing a dauer-constitutive phenotype (Vowels and Thomas 1994; Birnby *et al.* 2000). The gain-of-function nature of this allele is consistent with the results of numerous *hsp-90(RNAi)* experiments, including our own, that fail to show an increase in dauer formation (Piano *et al.* 2000; Rual *et al.* 2004; Gaiser *et al.* 2009, 2011; Waters *et al.* 2010; Melo and Ruvkun 2012; Frumkin *et al.* 2014). It is also consistent with our genetic analysis showing that *hsp-90(p673)* retains substantial HSP-90 activity and with the biochemical and biophysical analysis of bacterially-expressed wild-type and E292K (corresponding to *hsp-90(p673)*) *C. elegans* HSP-90 showing that this mutation only slightly reduces function of the protein (Gaiser *et al.* 2011). All of these data support the gain-of-function character of *hsp-90(p673)*. Specifically, Gaiser *et al.* (2011) demonstrated that the ATPase activity of the HSP-90(E292K) mutant protein is slightly reduced at 25°, but not at lower temperature. Moreover, the E292K substitution does not seem to alter the structural stability of the protein nor its binding of two HSP-90 co-chaperones, p23 (encoded by *ZC395.10*) and AHA-1, although binding to the STI-1 co-chaperone was slightly reduced (Gaiser *et al.* 2011).

With respect to *hsp-90(om40)*, Qiao *et al.* (1995) described a complex suite of phenotypes produced by *hsp-90(om40)*, leading the authors to conclude that *hsp-90(om40)* might have both loss- and gain-of-function features. For example, the Pro phenotype is less severe in *hsp-90(om40/null)* adults, a result usually interpreted to mean that a phenotype is a consequence of a gain-of-function. However, nearly all *hsp-90(om40)* phenotypes (Unc, arrested germline mitosis, delayed gametogenesis, oocyte defects, large germline nuclei, and sterility), including Pro, are recapitulated by creating *hsp-90(om40)* transheterozygotes with two severe loss-of-function alleles, *hsp-90(om118)* and *hsp-90(ok1333)* and by *hsp-90(RNAi)* knockdown (Piano *et al.* 2000; Rual *et al.* 2004; Gaiser *et al.* 2009, 2011; Waters *et al.* 2010; Frumkin *et al.* 2014). Thus, we conclude that *hsp-90(om40)*, which causes an I461N substitution, is a loss-of-function mutation and that the I461N substitution reduces HSP-90 activity.

HSP90 is assisted in its protein folding function of a plethora of clients by more than 20 different co-chaperones (Li *et al.* 2013; Schopf *et al.* 2017). Thus, the highly varied and complex phenotypes associated with different *hsp-90* alleles and with *hsp-90(RNAi)* are not surprising and are likely in part a consequence of disrupting both HSP-90’s interaction with any of a number of co-chaperones and the folding of at least several of the hundreds of client proteins with which HSP90 assists (https://www.picard.ch/downloads/Hsp90interactors.pdf). Furthermore, co-chaperones display a range of tissue expression patterns, including in *C. elegans*, suggesting that even wild-type HSP-90 activities will vary in different tissues (Haslbeck *et al.* 2012). This dizzying complexity of the HSP90 chaperone system may explain some of the unusual features of *hsp-90(om40)*. For example, a curious aspect of the *hsp-90(om40)* phenotype is a reversal of larval germline mitotic arrest and severe Unc defects when mutant animals reach late larval stages and adulthood (Qiao *et al.* 1995). Perhaps HSP-90(I461N) has impaired interactions with co-chaperones and/or client proteins expressed in larval germline and muscle, but has increased activity in older animals because of differences in co-chaperones and client proteins produced during late larval and adult stages.

In wild-type *C. elegans*, mitotic proliferation of germline stem cells is restricted to the distal region of the germline. However, one of the unusual loss-of-function phenotypes seen in *hsp-90(om40)* and *hsp-90(RNAi)* worms is proliferation in the proximal region of the germline (Pro phenotype) leading to formation of a proximal germline tumor. How does reduction of HSP-90 function, which appears to reduce GLP-1/Notch signaling and therefore reduce distal germline mitotic proliferation, paradoxically promote proximal germline mitotic proliferation? Whatever the mechanism, it seems to involve GLP-1/Notch signaling because proximal tumor formation is suppressed by the loss of *glp-1* function (Qiao *et al.* 1995). In *hsp-90(om40)* and *hsp-90(RNAi)* hermaphrodites, mitotic arrest during larval germline development delays the entry of germ cells into meiosis and exposes them to a proposed latent sheath cell niche, thereby stimulating their proliferation via GLP-1/Notch signaling, as described for mutations in several other genes (McCarter *et al.* 1997; Killian and Hubbard 2005; McGovern *et al.* 2009; Korta and Hubbard 2010). It is not clear, though, what the mechanism is for proximal tumor formation in males because males do not have gonad sheath cells (Wolf *et al.* 1978), but the numerous membrane-bound and secreted Notch ligands expressed in *C. elegans* are possible candidates for activating proximal proliferation in males as well as in hermaphrodites (Chen and Greenwald 2004; Komatsu *et al.* 2008; Greenwald and Kovall 2013). Further research into the HSP-90 chaperone system, including the roles and expression patterns of specific co-chaperones, should help to illuminate the complex functions of HSP-90 reported here.

## Supplementary Material

Supplemental Material is available online at www.g3journal.org/lookup/suppl/doi:10.1534/g3.118.300551/-/DC1.
