# Supplementary material for: The Molecular Chaperone HSP90 Promotes Notch Signaling in the Germline of Caenorhabditis elegans
Source: G3 (Bethesda). 2018 Mar 5;8(5):1535–44. doi: 10.1534/g3.118.300551 (PMC5940146; doi:10.1534/g3.118.300551)
Supplement: Supplementary file 1 [file 1535FileS1.docx]

**
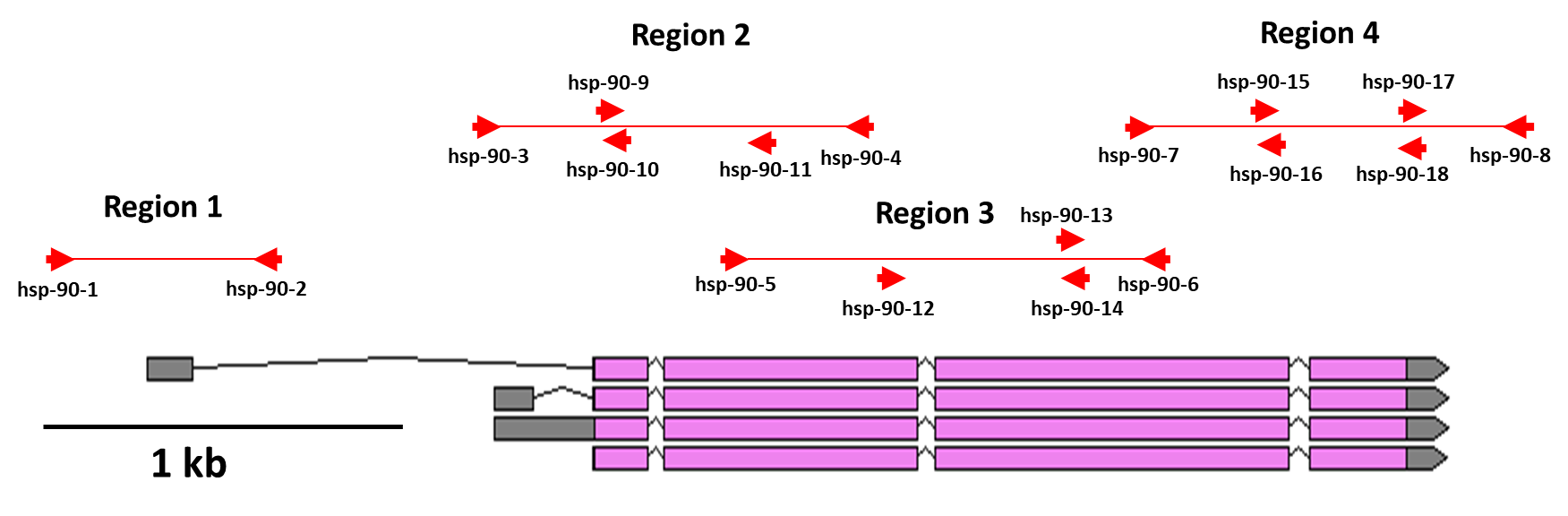
Figure S1 Locations of primers used to amplify and sequence regions of the *hsp-90* gene to identify mutations.**

Top, regions amplified by PCR are shown as lines labeled Region 1-4; primers are indicated with arrowheads (see Table S3). Bottom, the four predicted *hsp-90* transcript isoforms ([www.wormbase.org](http://www.wormbase.org)).

**
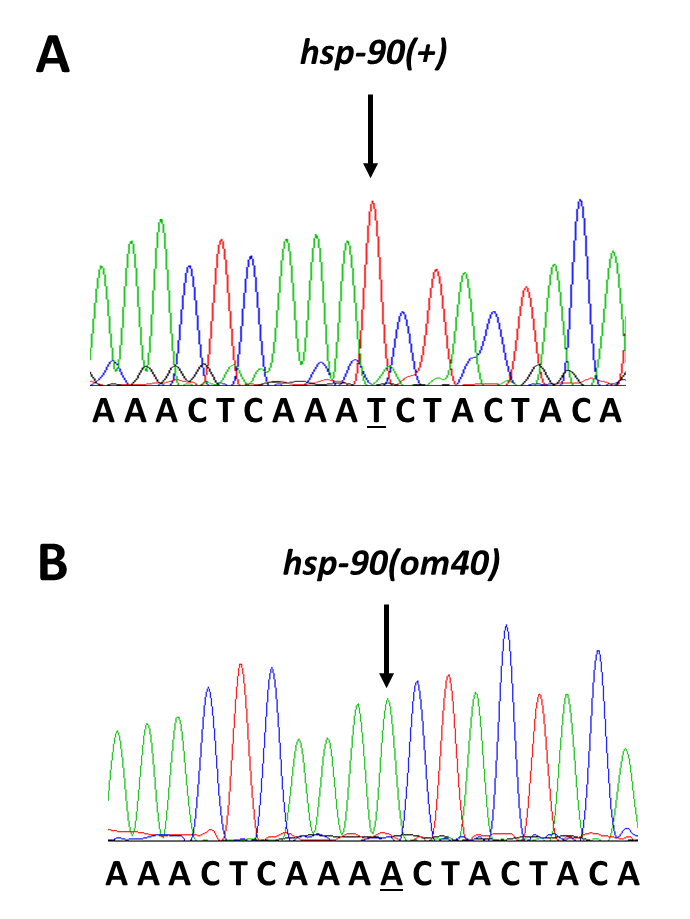
Figure S2 Sanger DNA sequencing chromatograms of *hsp-90(+)* and *hsp-90(om40)* PCR amplicons**

A. Partial chromatogram of Region 3 PCR amplicon from *hsp-90(+)* sequenced with primer hsp-90-13**.**

B. Partial chromatogram of Region 3 PCR amplicon from *hsp-90(om40)* sequenced with primer hsp-90-13.

Arrows indicated location of wildtype and mutant bases; wildtype and mutant bases are underlined in the sequence below each chromatogram.

**
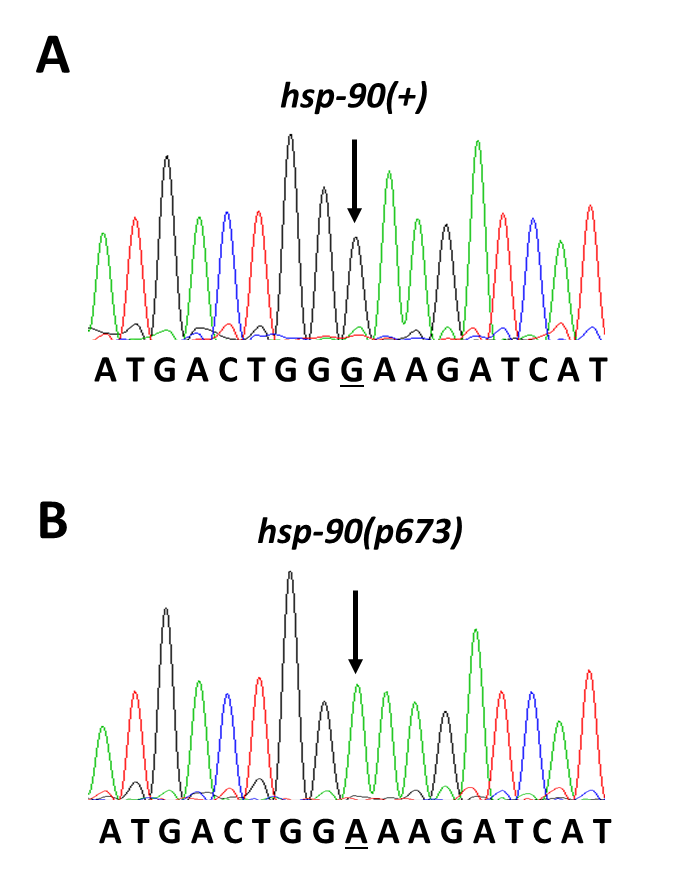
Figure S3 Sanger DNA sequencing chromatograms of *hsp-90(+)* and *hsp-90(p673)* PCR amplicons**

A. Partial chromatogram of Region 3 PCR amplicon from *hsp-90(+)* sequenced with primer hsp-90-12**.**

B. Partial chromatogram of Region 3 PCR amplicon from *hsp-90(om40)* sequenced with primer hsp-90-12.

**
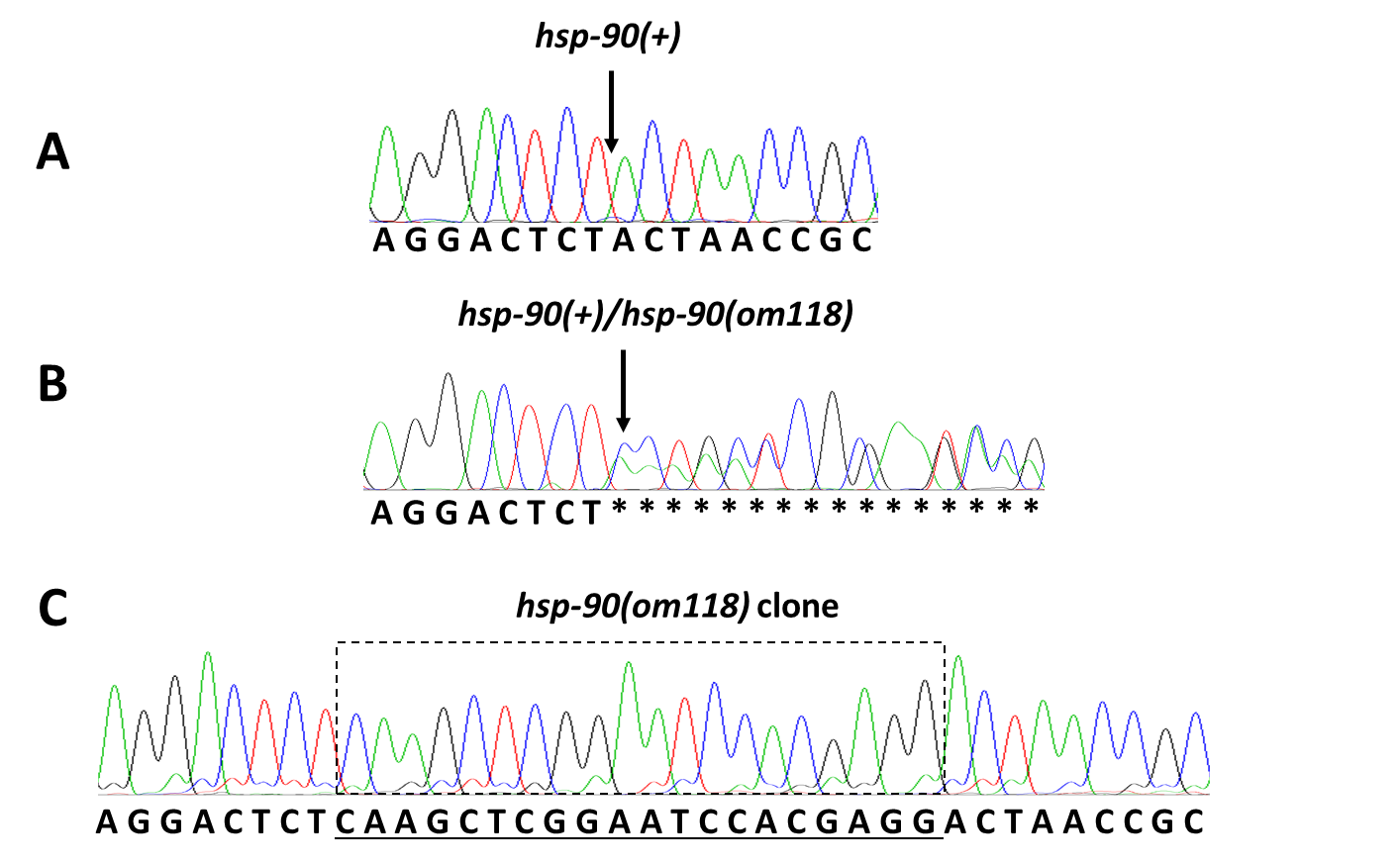
**

**Figure S4 Sanger DNA sequencing chromatograms of *hsp-90(+)* and *hsp-90(+)/hsp-90(om118)* PCR amplicons and *hsp-90(om118)* plasmid clone**

A. Partial chromatogram of Region 3 PCR amplicon from *hsp-90(+)* sequenced with primer hsp-90-13**.** Arrow indicates location of insertion in *hsp-90(om118).*

B. Partial chromatogram of Region 3 PCR amplicon from *hsp-90(+)/hsp-90(om118)* sequenced with primer hsp-90-13. Arrow indicates location of start of inserted sequence in *hsp-90(om118).* The nucleotide sequence is shown below; * overlapping sequence region.

C. Partial chromatogram of *hsp-90(om118)* plasmid clone sequenced with primer hsp-90-13. Dashed box shows inserted bases. The nucleotide sequence with the inserted sequence underlined is shown below.

**Table S1: Gonad transcriptome data for protein-coding genes in the *ego-3* region**

| **Gene / ORF** | **RPKM** |
| --- | --- |
| ***rfc-1*/ C54G10.2** | **47** |
| ***pmp-3*/ C54G10.3** | **125** |
| **C54G10.4** | **<1** |
| ***srh-17*/ C47E8.2** | **<1** |
| **C47E8.9** | **<1** |
| **C47E8.1** | **1** |
| **C47E8.3** | **<1** |
| **C47E8.4** | **248** |
| ***daf-21*/*hsp-90/*C47E8.5** | **2459** |
| **C47E8.11** | **24** |
| **C47E8.6** | **<1** |
| ***unc-112*/ C47E8.7** | **10** |
| ***set-5*/ C47E8.8** | **9** |
| **R08A2.9** | **NA** |

**Reference for the adult hermaphrodite gonad RNA-seq data is Guo *et al.* (2015). RPKM, reads per kilobase of transcript per million mapped reads.**

**Table S2 Codon and splicing changes detected in *ego-3(om40)* by whole genome sequence analysis**

| **Chr.** | **Nucleotide position** | **Nucleotide change** | | **ORF** | **Gene name** | **Reference sequence**  **amino acid** | ***ego-3(om40)***  **amino acid** |
| --- | --- | --- | --- | --- | --- | --- | --- |
| **I** | **200400** | **C** | **T** | **K10E9.1** | **K10E9.1** | **P** | **S** |
| **I** | **5672950** | **C** | **A** | **F55F8.9** | **F55F8.9** | **C** | **F** |
| **I** | **7092902** | **C** | **T** | **F22D6.5** | **prpf-4** | **A** | **T** |
| **I** | **7353179** | **C** | **G** | **F07A5.2** | **F07A5.2** | **P** | **A** |
| **I** | **8651317** | **G** | **A** | **F39H2.2** | **sig-7** | **R** | **K** |
| **I** | **8840853** | **C** | **G** | **F29D10.1** | **F29D10.1** | **Q** | **E** |
| **I** | **12901182** | **C** | **G** | **Y18D10A.13** | **pad-1** | **T** | **S** |
| **I** | **13484417** | **A** | **G** | **Y48G10A.2** | **Y48G10A.2** | **I** | **T** |
| **I** | **14334390** | **G** | **A** | **F49B2.6** | **F49B2.6** | **V** | **I** |
| **I** | **15053241** | **A** | **G** | **F31C3.5** | **F31C3.5** | **K** | **E** |
| **II** | **1678706** | **G** | **A** | **F58E1.7** | **F58E1.7** | **H** | **Y** |
| **II** | **2259158** | **G** | **T** | **K09F6.3** | **K09F6.3** | **E** | ***** |
| **II** | **2814088** | **G** | **A** | **K12H6.2** | **K12H6.2** | **S** | **F** |
| **II** | **2830577** | **G** | **A** | **K12H6.6** | **K12H6.6** | **H** | **Y** |
| **II** | **3503022** | **G** | **A** | **Y49F6B.2** | **Y49F6B.2** | **V** | **I** |
| **II** | **3614725** | **G** | **A** | **F40E12.2** | **F40E12.2** | **G** | **D** |
| **II** | **4196884** | **G** | **A** | **F31D5.5** | **F31D5.5** | **P** | **L** |
| **II** | **4321730** | **A** | **T** | **Y52E8A.1** | **Y52E8A.1** | **Y** | **F** |
| **II** | **4502831** | **G** | **A** | **T05C1.4** | **camt-1** | **S** | **F** |
| **II** | **4560537** | **G** | **A** | **M03A1.8** | **M03A1.8** | **G** | **E** |
| **II** | **10643270** | **C** | **T** | **M28.9** | **M28.9** | **P** | **L** |
| **II** | **11468119** | **T** | **G** | **W02B12.8** | **rga-1** | **L** | **R** |
| **II** | **11882797** | **G** | **C** | **ZK930.5** | **ZK930.5** | **R** | **T** |
| **II** | **12989293** | **T** | **C** | **Y38F1A.5** | **cyd-1** | **M** | **T** |
| **II** | **14310194** | **C** | **T** | **Y54G11A.13** | **ctl-3** | **G** | **D** |
| **II** | **14526832** | **C** | **T** | **F57C2.5** | **F57C2.5** | **R** | **H** |
| **II** | **14816511** | **C** | **T** | **W01D2.2** | **nhr-61** | **A** | **T** |
| **III** | **15852** | **G** | **T** | **H10E21.1** | **H10E21.1** | **D** | **Y** |
| **III** | **1711313** | **A** | **T** | **Y22D7AR.2** | **Y22D7AR.2** | **I** | **F** |
| **III** | **2309106** | **A** | **G** | **Y54F10BM.20** | **Y54F10BM.20** | **D** | **G** |
| **III** | **3845002** | **G** | **A** | **C36A4.5** | **C36A4.5** | **G** | **E** |
| **III** | **9939021** | **C** | **T** | **K03H1.5** | **K03H1.5** | **T** | **I** |
| **III** | **12023892** | **G** | **A** | **Y79H2A.1** | **brp-1** | **S** | **N** |
| **III** | **13398942** | **C** | **T** | **F11F1.1** | **F11F1.1** | **A** | **V** |
| **IV** | **1679148** | **G** | **A** | **Y41D4B.24** | **Y41D4B.24** | **T** | **I** |
| **IV** | **4946205** | **C** | **T** | **Y9C9A.4** | **str-169** | **P** | **L** |
| **IV** | **6521337** | **C** | **T** | **C01G5.9** | **C01G5.9** | **P** | **S** |
| **IV** | **10317918** | **C** | **T** | **R09E10.2** | **R09E10.2** | **E** | **K** |
| **IV** | **10793243** | **G** | **A** | **C06G8.2** | **pept-2** | **E** | **K** |
| **IV** | **10947707** | **C** | **T** | **Y69E1A.1** | **Y69E1A.1** | **V** | **I** |
| **IV** | **11157808** | **C** | **T** | **C08F8.4** | **mboa-4** | **R** | **H** |
| **IV** | **11818014** | **C** | **T** | **M117.1** | **M117.1** | **P** | **L** |
| **IV** | **12439546** | **C** | **T** | **C25G4.1** | **clec-185** | **S** | **L** |
| **IV** | **14379011** | **C** | **T** | **Y67A10A.7** | **Y67A10A.7** | **P** | **S** |
| **IV** | **15009066** | **C** | **T** | **Y41E3.4** | **qars-1** | **D** | **N** |
| **V** | **1494797** | **G** | **T** | **C38C3.10** | **C38C3.10** | **M** | **I** |
| **V** | **2157904** | **G** | **C** | **C45H4.8** | **srbc-22** | **Affects_Splicing** |  |
| **V** | **7472801** | **C** | **A** | **F25E5.12** | **str-28** | **A** | **S** |
| **V** | **8760428** | **T** | **C** | **B0507.10** | **B0507.10** | **K** | **E** |
| **V** | **10667640** | **T** | **C** | **F29F11.4** | **twk-12** | **Y** | **H** |
| **V** | **11646933** | **T** | **A** | **T27F2.1** | **skp-1** | **Y** | **N** |
| **V** | **13144537** | **G** | **T** | **B0365.7** | **dhc-3** | **S** | **I** |
| **V** | **14327786** | **A** | **T** | **F47B8.5** | **F47B8.5** | **V** | **D** |
| **V** | **14687640** | **T** | **A** | **C47E8.5** | **daf-21** | **I** | **N** |
| **V** | **14997197** | **C** | **A** | **T01C3.5** | **T01C3.5** | **Q** | **K** |
| **V** | **16102482** | **C** | **T** | **F22B8.6** | **cth-1** | **S** | **L** |
| **V** | **16163120** | **C** | **T** | **F10A3.11** | **F10A3.11** | **P** | **S** |
| **V** | **16252016** | **C** | **T** | **F21H7.9** | **gcy-20** | **A** | **T** |
| **V** | **19069825** | **G** | **A** | **Y39B6A.18** | **Y39B6A.18** | **P** | **L** |
| **X** | **4615858** | **C** | **T** | **T13H2.4** | **pqn-65** | **G** | **D** |
| **X** | **4677864** | **C** | **T** | **K02G10.4** | **flp-11** | **A** | **T** |
| **X** | **5785799** | **T** | **A** | **F46G11.3** | **tag-257** | **F** | **Y** |
| **X** | **5785800** | **T** | **G** | **F46G11.3** | **tag-257** | **F** | **L** |
| **X** | **7051613** | **T** | **G** | **B0403.2** | **ubc-17** | **F** | **V** |
| **X** | **8295255** | **G** | **A** | **R09F10.3** | **R09F10.3** | **G** | **E** |
| **X** | **9407264** | **C** | **A** | **C23F12.1** | **fln-2** | **Y** | ***** |
| **X** | **9694200** | **G** | **A** | **C43C3.2** | **npr-18** | **S** | **N** |
| **X** | **11077815** | **C** | **T** | **W04G3.10** | **W04G3.10** | **G** | **E** |
| **X** | **11830081** | **G** | **A** | **C34E11.3** | **tag-241** | **M** | **I** |
| **X** | **13619474** | **G** | **A** | **Y12A6A.2** | **Y12A6A.2** | **G** | **E** |
| **X** | **14248247** | **C** | **A** | **K04C1.5** | **K04C1.5** | **D** | **E** |
| **X** | **17459902** | **G** | **A** | **C36E6.3** | **mlc-1** | **M** | **I** |

**Reads were aligned to *C. elegans* reference genome WS230 and SNPs affecting protein-coding regions were identified. Nucleotide positions were then converted to the coordinates in WS238. The only variant identified within the genetically mapped region is highlighted in yellow.**

**Table S3 Primers used to amplify and sequence regions of the *hsp-90* gene to identify mutations**

| **Primer Name** | **Primer sequence** | **Description** |
| --- | --- | --- |
| **hsp-90-1** | **GTGTCGACAGTTGGGTCCAT** | **upstream primer to amplify and sequence region 1** |
| **hsp-90-2** | **ACAGTCGAAGAAGTCGTGAAGT** | **downstream primer to amplify and sequence region 1** |
| **hsp-90-3** | **CGCCCTCCTTCGAGAACATT** | **upstream primer to amplify and sequence region 2** |
| **hsp-90-4** | **TTCTTGTCAGCGTCGTCAGC** | **downstream primer to amplify and sequence region 2 AND reverse primer to sequence region 3** |
| **hsp-90-5** | **TGCCAAGTCTGGAACCAAGG** | **upstream primer to amplify and sequence region 3 AND forward primer to sequence region 2** |
| **hsp-90-6** | **TCCTTGAGTTGTTGGACGCA** | **downstream primer to amplify and sequence region 3** |
| **hsp-90-7** | **TCACCGGAGAGTCCAAGGAT** | **upstream primer to amplify and sequence region 4** |
| **hsp-90-8** | **GGACGTGGAGGTGGATTGAA** | **downstream primer to amplify and sequence region 4** |
| **hsp-90-9** | **AAACCTTCGCATTCCAGG** | **forward primer for sequencing region 2** |
| **hsp-90-10** | **CAATTCACGCAAGTAGATTTCC** | **reverse primer for sequencing region 2** |
| **hsp-90-11** | **CGGAAGACTCCCATTGATAG** | **reverse primer for sequencing region 2** |
| **hsp-90-12** | **GACCAAGAAAATCAAGGAGAAG** | **forward primer for sequencing region 3** |
| **hsp-90-13** | **CCGAGGACAAAGACAACTTC** | **forward primer for sequencing region 3** |
| **hsp-90-14** | **GGAAAGCTTCTTGCGGTTA** | **reverse primer for sequencing region 3** |
| **hsp-90-15** | **CGCATCATGAAAGCTCAAG** | **forward primer for sequencing region 4** |
| **hsp-90-16** | **ATGATAGCGTGGTCTGGGT** | **reverse primer for sequencing region 4** |
| **hsp-90-17** | **CATGGAGGAGGTCGACTAAA** | **forward primer for sequencing region 4** |
| **hsp-90-18** | **ATAAAACTGTGGAGGGGCTT** | **reverse primer for sequencing region 4** |
